# Functional assessment of the dural lymphatic vessels using dynamic contrast MRI in multiple sclerosis

**DOI:** 10.1002/brb3.3042

**Published:** 2023-05-22

**Authors:** Refaat E. Gabr, John A. Lincoln, Khader M. Hasan, Larry A. Kramer

**Affiliations:** ^1^ Departments of Diagnostic and Interventional Imaging University of Texas Health Science Center at Houston (UTHealth Houston) Houston Texas; ^2^ Departments of Neurology University of Texas Health Science Center at Houston (UTHealth Houston) Houston Texas

**Keywords:** cerebrospinal fluid, dural lymphatic vessels, dynamic contrast‐enhanced MRI, gadolinium‐based contrast agents, glymphatic function, multiple sclerosis

## Abstract

**Background and purpose:**

The discovery of glymphatic function in the human brain has generated interest in waste clearance mechanisms in neurological disorders such as multiple sclerosis (MS). However, noninvasive in vivo functional assessment is currently lacking. This work studies the feasibility of a novel intravenous dynamic contrast MRI method to assess the dural lymphatics, a purported pathway contributing to glymphatic clearance.

**Methods:**

This prospective study included 20 patients with MS (17 women; age = 46.4 [27, 65] years; disease duration = 13.6 [2.1, 38.0] years, expanded disability status score (EDSS) = 2.0 [0, 6.5]). Patients were scanned on a 3.0T MRI system using intravenous contrast‐enhanced fluid‐attenuated inversion recovery MRI. Signal in the dural lymphatic vessel along the superior sagittal sinus was measured to calculate peak enhancement, time to maximum enhancement, wash‐in and washout slopes, and the area under the time‐intensity curve (AUC). Correlation analysis was performed to examine the relationship between the lymphatic dynamic parameters and the demographic and clinical characteristics, including the lesion load and the brain parenchymal fraction (BPF).

**Results:**

Contrast enhancement was detected in the dural lymphatics in most patients 2–3 min after contrast administration. BPF had a significant correlation with AUC (*p* < .03), peak enhancement (*p* < .01), and wash‐in slope (*p* = .01). Lymphatic dynamic parameters did not correlate with age, BMI, disease duration, EDSS, or lesion load. Moderate trends were observed for correlation between patient age and AUC (*p* = .062), BMI and peak enhancement (*p* = .059), and BMI and AUC (*p* = .093).

**Conclusion:**

Intravenous dynamic contrast MRI of the dural lymphatics is feasible and may be useful in characterizing its hydrodynamics in neurological diseases.

## Graphical abstract

We study the feasibility of a novel intravenous, dynamic, contrast‐enhanced MRI method to assess the dural lymphatic vessels. Initial results in multiple sclerosis patients confirm feasibility, and show interesting associations between the lymphatic signal and patient characteristics. The proposed functional method could enable in vivo study of waste clearance mechanisms in neurological disorders.

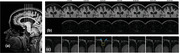

## 1 INTRODUCTION

Lymphatic vessels have historically been thought of as absent in the CNS. However, the recent discovery of a CNS macroscopic waste clearance mechanism, referred as the glymphatic system, has challenged this notion (Jessen et al., 2015). In distinction to lymphatic vessels, the glymphatic system is thought to clear fluid and solute waste through an astrocyte‐mediated transport pathway (Dupont et al., 2019; Iliff et al., 2012). In this model, cerebrospinal fluid (CSF) enters the brain parenchyma through periarterial spaces, mixes with the interstitial fluid, and reenters the subarachnoid space via the perivenular spaces, carrying with it parenchymal waste. From here, subarachnoid CSF absorption is linked to the cervical lymphatics in both human and a multitude of animal studies (Albayram et al., 2022; Koh et al., 2005; Louveau et al., 2018). In contrast, the classic theory that arachnoid projections (villi and granulations) are potential sites of significant CSF efflux into the venous sinuses lacks current support (Proulx, 2021) underscoring the importance of lymphatic outflow pathway in CNS homeostasis.

The putative glymphatic system has been assessed by imaging the redistribution of intrathecally administrated contrast agent within the human brain with follow‐up imaging of 24–48 h after contrast injection (Eide et al., 2018; Eide et al., 2021; Ringstad et al., 2017; Watts et al., 2019; Zhou et al., 2020). However, intrathecal contrast injection methodology has limited clinical application due to the risks associated with a lumbar puncture and the extensive imaging period required. Thus, intravascular administration of contrast agents and several hours of delay have been investigated as a less‐invasive alternative (Deike‐Hofmann et al., 2019; Lee et al., 2021).

Meningeal lymphatic vessels have recently been imaged in vivo in healthy human and nonhuman primates (Absinta et al., 2017; Albayram et al., 2022; Ringstad & Eide, 2020; Wu et al., 2021). These lymphatic vessels are specifically located within the dura mater and highly concentrated along the superior sagittal sinus (Visanji et al., 2018). In a mouse model using direct brain parenchymal injection of an inert tracer, it has been shown that dural lymphatic vessels absorb CSF from adjacent subarachnoid space and brain interstitial fluid via the meningeal lymphatic system (Aspelund et al., 2015). A potentially much less‐invasive way to trace glymphatic clearance is by monitoring the dural lymphatics following intravenous contrast administration. Dural lymphatic vessels enhance after intravenous contrast injection due to partial diffusion of contrast from dural blood vessels into the adjacent dural lymphatic vessels (Absinta et al., 2017).

An additional important property of the meningeal lymphatic system is that it contains a sizable portion of sinusal T cells and antigen‐presenting cells suggesting that they function in trafficking both meningeal and CSF contained immune cells (Louveau et al., 2018). Macromolecule accumulation in the meninges has been demonstrated in murine K14‐VEGFR3‐Ig transgenic mouse, a model lacking lymphatic vessels (Aspelund et al., 2015). Furthermore, resection of deep cervical lymph nodes, that serve as meningeal lymphatic drainage, resulted in less severe disease progression (Louveau et al., 2018; van Zwam et al., 2009). These findings have heightened the importance of further exploration of possible secondary mediators of MS pathology associated with the lymphatic function.

In this work, we study the feasibility of using dynamic intravenous contrast‐enhanced MRI to provide a rapid and less‐invasive functional assessment of the dural lymphatics as a potential methodology to study glymphatic clearance. This is achieved by monitoring the signal in the dural lymphatic vessels using high‐resolution MRI during a short period (∼30 min) after contrast injection.

## 2 METHODS

### 2.1 Study subjects

The study included 20 patients with a clinical diagnosis of MS according to 2017 McDonald Criteria (Thompson et al., 2018). The patient demographics and clinical characteristics are listed in Table 1. This study was conducted under approval of our Institutional Review Board, and written informed consent was obtained from all participants.

**TABLE 1:** Characteristics of the study participants.

Number of subjects	20
Females/males	17/3
Age (years), mean [range]	46.4 [27, 65]
BMI (kg/m^2^), mean [range]	25.9 [17.1, 35.9]
MS phenotype	
Relapsing‐remitting MS	13
Secondary‐progressive MS	7
Disease duration (years), mean, [range]	13.6 [2.1, 38.0]
EDSS (median, [range])	2.0 [0, 6.5]
Lesion load (mL), median [range]	3.9 [0.43, 75.1]
Brain parenchymal fraction, mean [standard deviation]	0.81 [0.67, 0.92]

EDSS: Expanded Disability Status Scale; BMI: body mass index.

### 2.2 MRI experiments

MRI was performed on a 3.0 Tesla Ingenia MRI scanner (Philips Healthcare, Best, The Netherlands) using a 32‐channel head coil. A standard MS imaging protocol was augmented to image the meningeal lymphatic vessels using a coronal T2‐weighted fluid‐attenuated inversion recovery (T2‐FLAIR) sequence, which is sensitive for the detection of gadolinium enhancement in the dural lymphatic vessels (Absinta et al., 2017). T2‐FLAIR has several important characteristics suitable for this purpose. It nulls the CSF signal while maintaining contrast enhancement due to a mild T1 relaxation effect and suppresses dural sinus signal from a combination of T2 signal loss relative to concentrated intraluminal gadolinium and the flow‐void phenomenon associated with T2‐FLAIR (Iancu‐Gontard et al., 2003; Lee et al., 2016). These inherent properties maximize detection of enhancing dural lymphatics near the dural sinus and subarachnoid space. T2‐FLAIR imaging was acquired once before and seven times after the administration of the gadolinium‐based contrast agent gadoterate meglumine (Dotarem, Guerbet, France) at dose of 0.1 mmol/kg injected at the rate of 2 mL/s. The T2‐FLAIR scan was acquired immediately before contrast administration, and repeated seven times postcontrast at a delay of 2.5, 5, 7, 9, 17, 20, and 35 min without retuning between any of the scans. The T2‐FLAIR scan used a turbo spin echo sequence with the following parameters: field of view = 37.8 × 37.8 mm; slice thickness = 3 mm; number of slices = 10; voxel size = 0.52 × 0.52 mm (reconstructed to 0.17 × 0.17 mm); repetition time/echo time/inversion time = 3000/90/1200 ms; echo train length = 21; refocusing angle = 110°, number of signal averages = 2; scan duration/acquisition = 126 s.

### 2.3 Image analysis

Regions‐of‐interest (ROIs) were placed in all slices to measure the signal from the dural lymphatic vessel inferior to the superior sagittal sinus. The inferior lymphatic vessel was specifically selected since it has a distinct location between the inferior most edge of the superior sagittal sinus and the falx cerebri that is reproducible across subjects. Additionally, compared to the more superior located lymphatic vessels lateral to the superior sagittal sinus, the inferior lymphatic vessel is largely bordered by avascular subarachnoid CSF and thereby more consistently isolated from surrounding enhancing soft tissues. Although contrast enhancement of the subarachnoid CSF has been described with the T2‐FLAIR sequence in pathologies associated with blood brain barrier breakdown resulting in contrast leakage, detection in the subarachnoid space typically occurs 1–24 h after intravenous administration (Bozzao et al., 2003) and therefore would not likely contaminate the ROI measurement within the time frame of data collection of this study. The vessel location was manually identified and a rectangular ROI was automatically placed centered at the user selection. The lymphatic signal at each time point was obtained from the robust maximum (98th percentile) of all voxel intensities at that time point, and normalized to the median brain signal in the precontrast scan.

**FIGURE 1:**
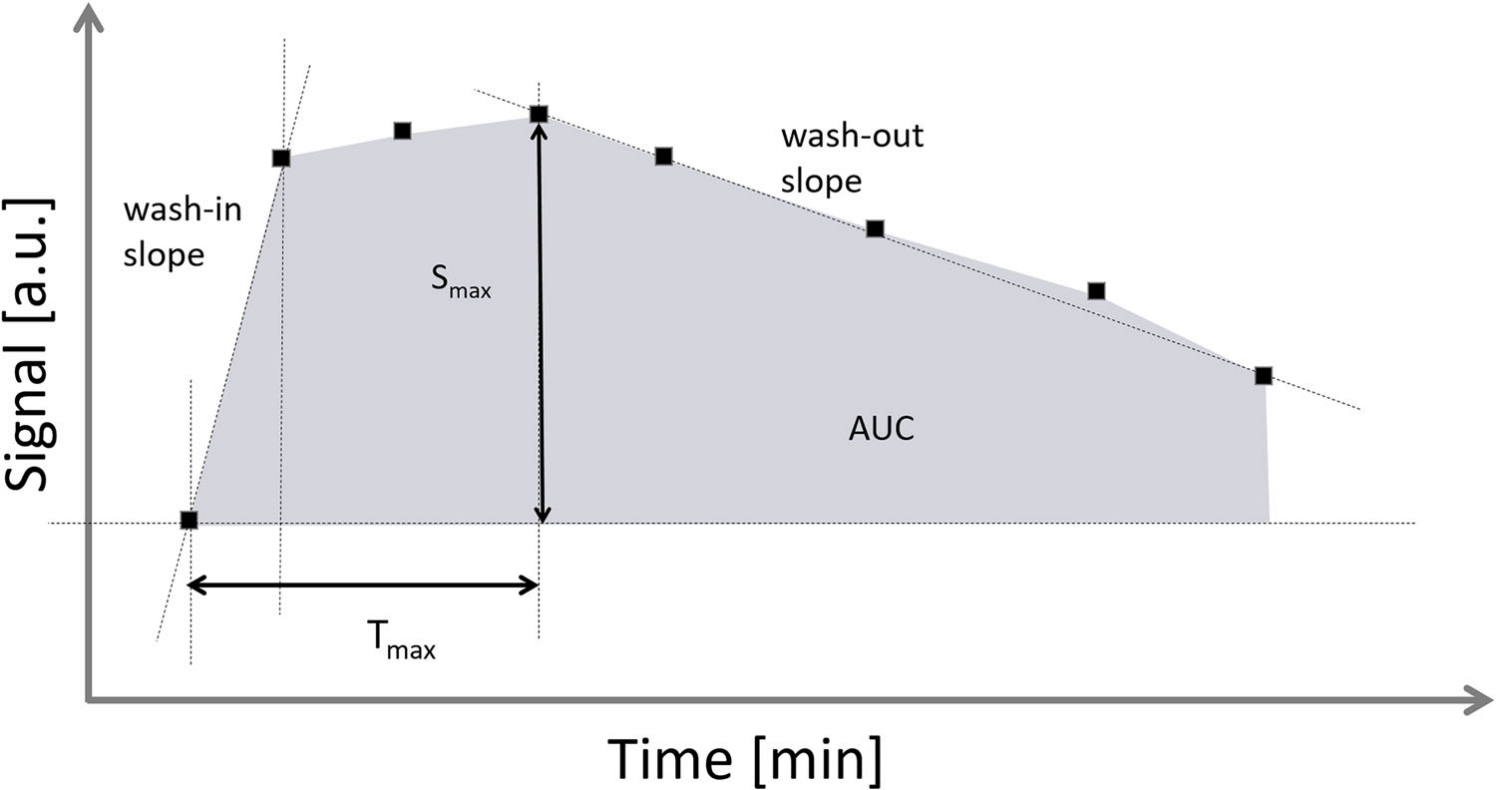
Parameters characterizing the dural lymphatic signal dynamics. *S*_max_: maximum signal in the lymphatic vessels; *T*_max_: time to maximum signal; AUC: area under the time‐signal curve; min: minutes; a.u.: arbitrary units.

To characterize the temporal dynamics of the lymphatic enhancement, the following metrics were computed from the lymphatic signal (Figure 1): time to peak signal (*T*_max_), peak enhancement *P*_enh_ = 100 × (*S*_max_ – *S*_0_)/*S*_0_, area under the time‐signal curve (AUC), wash‐in slope = (*S*_1_ – *S*_0_)/(*T*_1_ – *T*_0_), and washout slope = (*S*_max_ – *S*_last_)/(*T*_last_ – *T*_max_). Here *S* denotes the maximum lymphatic signal, *T* denotes the postcontrast delay, and the subscripts *S*_0_, S_1_, *S*_last_, and *S*_max_ denote the signal before contrast injection, first acquisition after contrast injection, last postcontrast acquisition, and the acquisition with peak enhancement.

### 2.4 Statistical analysis

Linear regression analysis was performed to explore the association between the glymphatic parameters and the patient age, body mass index (BMI), disease duration, the expanded disability status score (EDSS), T2 lesion load (T2LV, with cube root transformation; Li et al., 2006), and the brain parenchymal fraction (BPF). Brain tissue and T2 lesions were segmented using the volBrain software (Manjón & Coupé, 2016), and T2 lesions were manually reviewed/corrected by an experienced rater (JAL). A *p*‐value < .05 was considered statistically significant. Regression analysis was performed using the SciPy python module (version 1.7.1, scipy.org).

## 3 RESULTS

**FIGURE 2:**
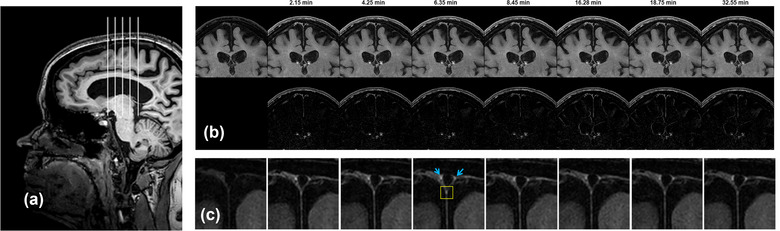
(A) Sagittal T1 images showing the location of the coronal contrast‐enhanced fluid‐attenuated inversion recovery (T2‐FLAIR) images. (B, upper row) Coronal FLAIR images before and at multiple time points (in minutes; [min]) after contrast administration in a 53‐year‐old female patient with relapsing‐remitting MS. (B, lower row) difference images relative to the precontrast scan. (C) Zoomed view showing the region of interest where contrast enhancement in the inferior dural lymphatic vessel (yellow box) was measured. The more superior dural lymphatic vessels are indicated by blue arrows. Note the excellent suppression of dural sinus and cerebrospinal fluid signal throughout the data collection period characteristic of the T2‐FLAIR sequence.

**FIGURE 3:**
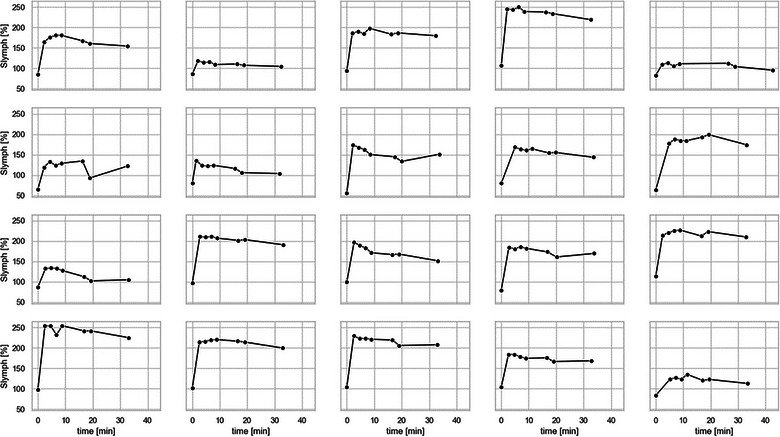
The normalized lymphatic signal as a function of time in minutes [min] after contrast administration in all 20 subjects. *S*_lymph_: signal in the dural meningeal lymphatic vessels expressed as percentage change from the precontrast brain signal.

The mean time delay for the seven acquisitions after contrast administration was (mean ± standard deviation): 2.76 ± 1.03, 4.86 ± 1.03, 6.96 ± 1.02, 9.06 ± 1.02, 17.09 ± 2.24, 19.62 ± 2.24, 33.40 ± 2.21 min. Figure 2 shows the acquired T2‐FLAIR images in one subject demonstrating contrast enhancement in the parasagittal lymphatic vessels. The signal time course in the lymphatic vessel in all subjects is shown in Figure 3. Sharp increase in the lymphatic signal was observed after contrast administration in most subjects, reaching a maximum at ∼7 min. This was followed by a slow decay of the signal by an average of ∼13% of the peak signal at the last acquisition.

**FIGURE 4:**
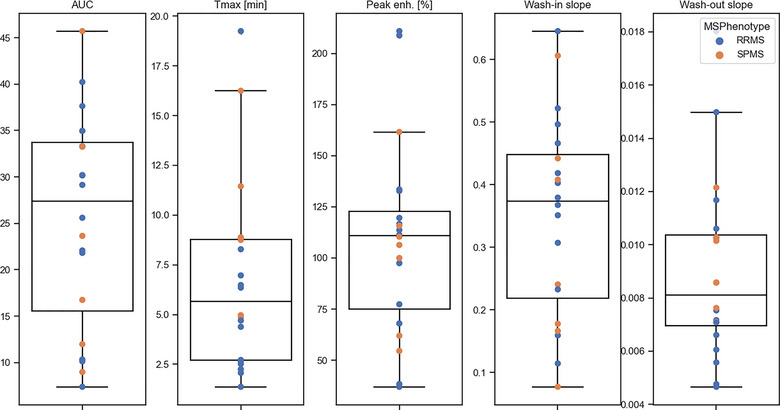
The measured lymphatic parameters. Peak enh.: percent maximum signal in the dural lymphatic vessels; *T*_max_: time to maximum signal in minutes [min]; AUC: area under the time‐signal curve. RRMS: relapsing‐remitting MS; SPMS: secondary‐progressive MS.

The measured parameters of the dynamic contrast enhancement for all subjects are shown in Figure 4. The average peak enhancement was 109% ± 47% and the time to peak enhancement was 6.70 ± 4.72 min. The wash‐in slope was 0.35 ± 0.16 min^−1^ and the washout slope was 0.009 ± 0.0034 min^−1^. The AUC was 25.42 ± 11.44 min.

**FIGURE 5:**
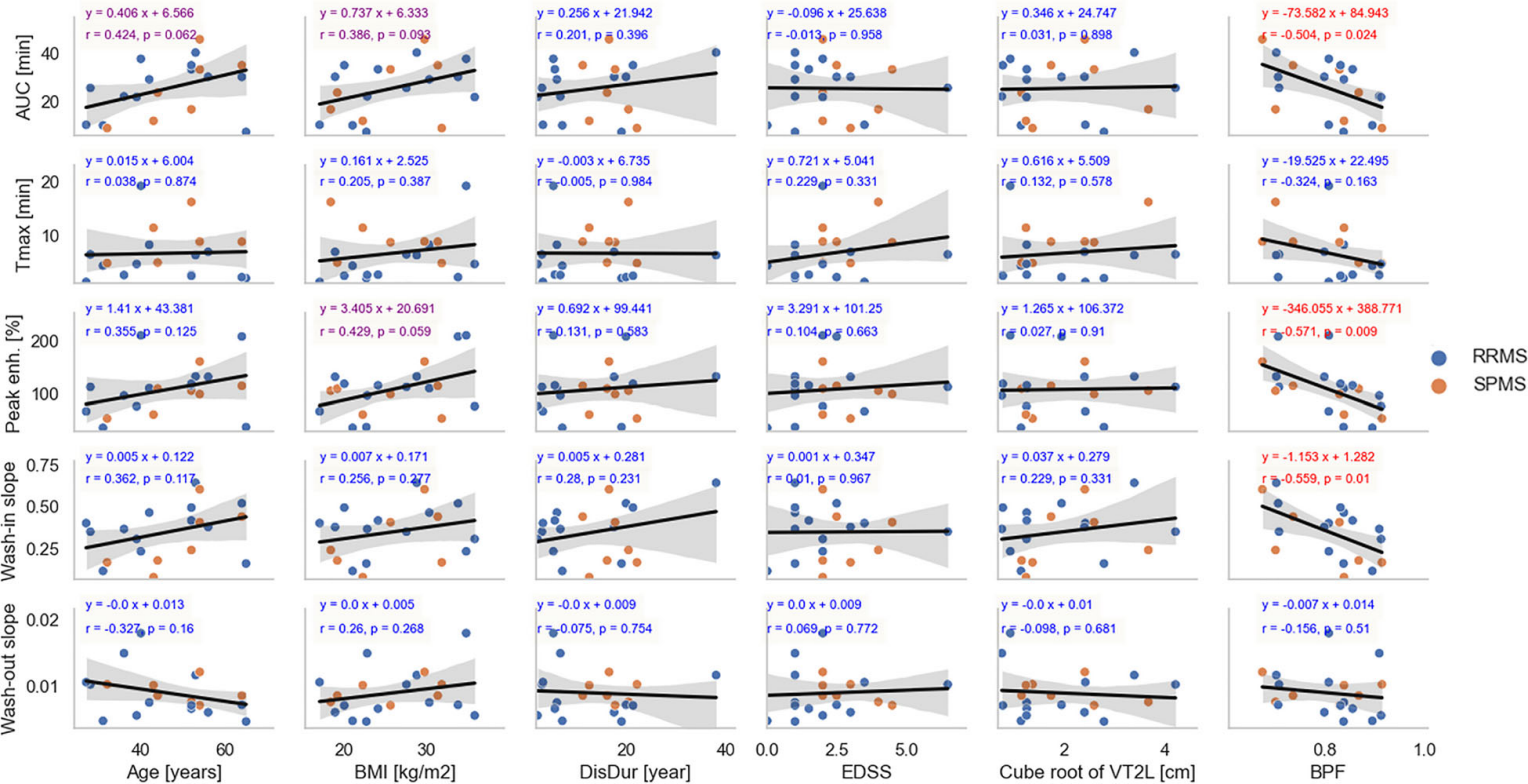
Regression analysis between the parameters characterizing the lymphatic dynamics (rows) and the patient demographic and clinical measures (columns). Peak enh.: percent maximum signal in the lymphatic vessels; *T*_max_: time to maximum signal in minutes [min]; AUC: area under the time‐signal curve; BMI: body mass index; EDSS: expanded disability status scale; DisDur: disease duration; V2TL: volume of T2 lesions; BPF: brain parenchymal fraction. Equations of best‐fit lines are shown and colored according to *p*‐values (red, *p* < .05; magneta, 0.05 ≤ *p* < .1; blue, *p*≥0.1). RRMS: relapsing‐remitting MS; SPMS: secondary‐progressive MS.

Correlations between the parameters of the lymphatic signal and the patient demographic and clinical measures are shown in Figure 5. BPF correlated with AUC (*r* = −0.50, *p* < .03), peak enhancement (*r* = −0.57, *p* < .01), and the wash‐in slope (*r* = −0.56, *p* = .01). No statistically significant correlations were found between the patient age, BMI, disease duration, EDSS, or lesion load and the lymphatic signal parameters. However, a trend was found between the patient age and AUC (*r* = 0.42, *p* = .062). Trends were also observed between the patient BMI and both peak enhancement (*r* = 0.42, *p* = .059) and AUC (*r* = 0.39, *p* = .093). Due to the small sample size, no analysis was performed for sex or ethnicity correlations.

## 4 DISCUSSION

In this work, we used dynamic intravenous contrast MRI to detect and characterize passage of the contrast agent through the dural lymphatic vessels in MS patients on a standard 3T MRI system. Contrast enhancement was detectable in the lymphatic vessels, and the signal was tracked for ∼33 min after contrast injection with 109% average peak enhancement.

Due to the invasiveness of intrathecal contrast injection in glymphatic imaging studies, less‐invasive alternatives were developed using intravascular administration of contrast agents. For example, intravenous contrast was used with a long delay of 3 and 24 h to study signal intensity changes in various cerebral fluid spaces and white matter hyperintensities (Deike‐Hofmann et al., 2019). T1 mapping was additionally used to monitor contrast distribution in the brain at baseline and up to 12 h after intravenous contrast injection (Lee et al., 2021). Our pilot study extends the application of intravascular contrast methods to the study of the recently discovered human brain meningeal lymphatic vessels (Absinta et al., 2017) using dynamic contrast‐enhanced MRI. This approach can produce new lymph vessel‐specific information that is complementary to the information obtained from contrast redistribution in the CSF and brain parenchyma. Together, these techniques may enable a more complete picture of the status of the putative glymphatic function.

The proposed approach for imaging the lymphatic vessels enjoys the benefit of a short scan time, which can be conducted in the same scan session, making it more compatible with clinical imaging protocols. While the proposed dynamic study is short compared to other studies addressing the brain glymphatic function, the follow‐up time of 33 min is still considerably long. The scan time however may be used more efficiently by acquiring other sequences in between the lymph imaging acquisitions, taking advantage of the identified slow washout rate. In this work, for example, postcontrast 3D T1w (MP‐RAGE) was acquired after the first four lymphatic dynamics, allowing good capture of the fast wash‐in dynamics of the lymphatic signal, and steady state contrast‐enhanced T1w imaging. In addition, high‐resolution, whole‐brain FLAIR scan images were acquired between the 6th and the 7th dynamics.

The primary aim of this study was to demonstrate technical feasibility of performing functional assessment of the dural lymphatic vessels in a clinically compatible MRI study. To further show the possible clinical utility of the proposed approach, associations between the lymphatic signal dynamics and the patients’ age, BMI, and disease characteristics were evaluated. BPF, a measure of brain atrophy, was found to correlate with three measures of the lymphatic enhancement (AUC, peak enhancement, and wash‐in slope). These altered enhancement parameters with BPF may indicate impeded flow in the dural lymphatics as a result of MS pathology combined with age‐related cervical lymph node atrophy and dural lymphatic channel thickening (Albayram et al., 2022) and/or hyperpermeability of capillaries associated with aging (Oakley & Tharakan, 2014) increasing the transfer rate of contrast material to the dural lymphatics. We did not find statistically significant correlations with the clinical measures of disease duration, EDSS, or lesion load in our MS cohort, likely due to the small sample size and disease heterogeneity. However, certain age and BMI trends were identified. AUC trended with increasing age and higher BMI. Similarly, peak enhancement trended with BMI. These observations suggest that the lymphatic dynamics may be sensitive for the detection of altered hemodynamics in severely diseased, older, and/or obese subjects. These results are indeed in line with a recent study, which showed lower diffusion along the perivascular space in MS patients associated with grey matter atrophy (Carotenuto et al., 2022). Prior studies similarly demonstrated impaired glymphatic clearance in older mice (Kress et al., 2014) and impaired integrity of basal meningeal lymphatic vessel and CSF drainage with aging in mice (Ahn et al., 2019). Age was also an independent factor for delayed clearance of the glymphatic pathway in aging humans (Zhou et al., 2020). In addition, BMI is a surrogate for cardiovascular risk in patients and has been shown to be independently associated with worsening cognitive performance and clinical progression (Castro et al., 2019; Filippatou et al., 2020; Manouchehrinia et al., 2018; Owji et al., 2019). Both age and BMI were also associated with higher ratio of cerebral perivascular spaces volume ratio in healthy humans (Barisano et al., 2020). Unlike previous work (Carotenuto et al., 2022), we did not find correlation between the lymphatic parameters and the lesion load.

Several studies have championed the intertwined concepts of cerebrovascular dysfunction as contributing to neurodegenerative aspects of the pathogenesis of MS (Haider et al., 2016; Mahad et al., 2015; Trapp & Stys, 2009). A recent large retrospective study showed that higher cardiovascular risk was associated with an increased risk of MS relapse and worsening disability (Petruzzo et al., 2021). Animal models have suggested an important role for the glymphatic system in the removal of macromolecular waste, subpial immune aggregation and disease progression (Absinta et al., 2017; Hsu et al., 2019; Louveau et al., 2018; van Zwam et al., 2009). However, in vivo mechanistic studies evaluating the role of lymphatic health or the putative glymphatic function in MS are lacking. The proposed approach could enable the future investigation of this role using a less‐invasive and clinically compatible imaging protocol compared to existing methods.

Heterogeneity was observed in the lymphatic enhancement patterns of the study patients (Figure 3). For example, few patients show substantially weaker contrast enhancement in the lymphatic vessels compared to the other (Figure 3, row 1, column 2; row 1, column 5; row 4, column 5). These three cases correspond to 65‐year‐old Caucasian female with RRMS, 31‐year‐old Caucasian female with RRMS, 43‐year‐old African American female with SPMS. We observed interesting associations between the lymphatic parameter and patient demographics (sex, ethnicity) and disease phenotype. However, the sample size in the current study was too small to allow a reliable statistical analysis. The reasons for the heterogeneity in the lymphatic enhancement patterns are thus not completely understood at this stage, and further investigation with a larger study is warranted.

The time scale for dynamic signal enhancement of dural lymphatics was another unknown. Hence, one of the goals of this work was to identify that time scale. We targeted a relatively long follow‐up duration that could still be tolerated by the study participants. Approximately 30 min was deemed clinically feasible. In addition, the sampling frequency was another unknown and we designed the experiments to have a relatively high temporal resolution during the wash‐in period. At later time points where we expected a slower rate of change, we sampled more sparsely to limit unnecessary radio‐frequency exposure to the participants and to reduce the redundancy in the collected data. Variations in the measurement time are hard to completely control in clinical settings since this depends on patient compliance. Good compliance is particularly required in the fast wash‐in period, for which we had a variation of ∼1 min. At later time points, however, variations in the time delay, here ∼2 min, are not expected to substantially affected the calculated parameters due to the slower contrast dynamics.

This feasibility study was limited by the small number of subjects and the heterogeneity of MS, making it impractical to conduct several other statistical analyses. Future larger studies are needed to assess the effects of age, sex, ethnicity, MS phenotype and to study the relationships between the lymphatic parameters and other imaging and clinical measures. Another limitation was the absence of control healthy subjects in whom contrast administration may not be sufficiently justified with no prior evidence on its potential usefulness in assessing meningeal lymphatic vessels. With the promising potential of the proposed method, future work will include healthy subjects to determine normative values for the lymph vessel dynamics and to assess whether the measured lymphatic metrics in MS are different from the normative values. Finally, the high‐resolution imaging protocol was limited to a small section of the brain to enable fine temporal sampling of the wash‐in period. Fast, whole‐brain, high‐resolution imaging methods are crucial to successfully extend the analysis to the whole lymphatic network.

In conclusion, we have demonstrated the feasibility of measuring and characterizing the temporal dynamics of the lymphatic vessels using intravenous contrast agents on widely available standard 3T MRI. The approach described could be integrated in routine MRI protocol to assess the dural lymphatic function and identify possible contributions to the pathophysiology of MS and other neurodegenerative disorders and as a methodology to evaluate novel treatments.

## CONFLICT OF INTEREST STATEMENT

The authors declare no conflict of interest.

### PEER REVIEW

The peer review history for this article is available at https://publons.com/publon/10.1002/brb3.3042.

## Data Availability

The data analyzed in this study are available upon reasonable request from the corresponding author.
